# Effects of wearable technology on mental engagement and physical activity participation: self-regulatory motivation, attitude, flow, and dropout in new sport participation

**DOI:** 10.3389/fpubh.2026.1911509

**Published:** 2026-08-12

**Authors:** So-Jung Park, Hye Jin Yang, Chulhwan Choi, Chul-Ho Bum

**Affiliations:** 1 Department of Physical Education, Graduate School, Kyung Hee University, Yongin-si, Gyeonggi-do, Republic of Korea; 2 Department of Physical Education, Gachon University, Seongnam-si, Gyeonggi-do, Republic of Korea; 3 College of Physical Education, Kyung Hee University, Yongin-si, Gyeonggi-do, Republic of Korea

**Keywords:** mental engagement, motivation, new participants, physical activity, wearable device

## Abstract

**Background:**

Wearable devices have emerged as promising tools for monitoring physical activity and supporting health management, yet their psychological effects on individuals newly engaged in sport participation remain underexplored.

**Objective:**

This study aimed to examine differences in self-regulatory motivation, participation attitude, flow, and dropout according to wearable device usage patterns among new sport participants.

**Methods:**

Using a cross-sectional survey design, data were collected from 226 new sport participants in South Korea, categorized into three groups by wearable device usage patterns: sharing-and-using, using-only, and non-using. Multivariate analyses of variance with *post-hoc* tests were conducted.

**Results:**

The wearable device usage groups demonstrated higher intrinsic and extrinsic motivation, lower amotivation, and higher perseverance, cognitive flow, and behavioral flow than the non-using group, whereas proactiveness showed no significant difference. Regarding dropout, only the using-only group demonstrated significantly lower levels than the non-using group.

**Conclusion:**

These findings suggest that wearable devices can positively influence the psychological experiences and exercise participation of new sports participants. However, the data sharing function could lead to negative experiences, such as social comparison and psychological burden. Therefore, to maximize the effectiveness of wearable devices, tailored utilization strategies that consider individual user characteristics and exercise experience levels are necessary.

## 1 Introduction

The importance of personal health management has been increasingly emphasized due to the decline in physical activity and the rise of stress and anxiety, which together contribute to the deterioration of both physical and psychological wellbeing (1). Physical inactivity has been identified as a major public health concern, contributing to increased risks of chronic disease, mental health disorders, and premature mortality worldwide (2). In particular, rising levels of stress and anxiety have been closely linked to reduced physical activity participation, creating a bidirectional relationship that further undermines both physical and mental wellbeing (3). These changes have highlighted the need for individuals to recognize and manage their own health conditions and physical activity levels. Accordingly, there is a growing demand for systematic self-management approaches that go beyond mere sports participation, enabling individuals to continuously monitor and regulate their physical activity and health status. With the advancement of information and communication technology, the ubiquitous era—in which information can be accessed regardless of time and place—has become a reality, making it easier for individuals to collect and manage personal health data (4). This technological progress has led to the widespread adoption of wearable devices, which allow for the continuous recording and management of health and activity information (5).

A wearable device is a compound term derived from “wearable” and “device,” referring to an Internet of Things-based apparatus worn on the body that monitors biosignals in real time (6). It is capable of automatically measuring and tracking a wide range of health information, including blood pressure, physical activity levels, and respiration (7). It also serves to enhance the efficiency of personal health management through self-tracking (7). Furthermore, owing to their connectivity with smartphones, wearable devices are being utilized across diverse fields, including healthcare, pharmaceuticals, fitness, and business management (8–10). Wearable devices vary considerably in their form and functionality. Smartwatches, for instance, offer screen-based interfaces and smartphone connectivity, enabling features such as messaging and data sharing. In contrast, basic fitness trackers and smart bands typically focus on activity monitoring without screen interfaces or social sharing capabilities (11). In the present study, wearable devices refer specifically to devices that automatically record and store physical activity or health-related data, including smartwatches, smart bands, and fitness trackers. Given these characteristics, the wearable device market is rapidly growing, particularly centered on fitness trackers and healthcare applications (6), and has established itself as a key healthcare tool (12). Global wearable device usage has expanded significantly in recent years, with the number of connected wearable devices worldwide estimated to exceed one billion units (13).

Although a growing body of research has examined the effects of wearable devices, most studies have focused primarily on technology acceptance factors or changes in health indicators (14, 15). Technology acceptance research has largely drawn on the Unified Theory of Acceptance and Use of Technology [UTAUT; (16)], which identifies performance expectancy, effort expectancy, social influence, and facilitating conditions as key determinants. Lee et al. (17) analyzed the influence of technological and personal characteristics of wearable devices on usage intention based on the UTAUT model, while An (18) confirmed the effect of increased physical activity levels through wearable device-based activity analysis (19). In particular, several studies have begun to focus on the effects of wearable devices beyond simple increases in physical activity, examining their influence on social interaction, motivation enhancement, and the maintenance of exercise adherence. Chang et al. (20) reported that sharing activity data via wearable devices promotes social support, thereby increasing users' intention to continue exercising and their actual levels of physical activity. They further identified the sharing function of wearable devices as a key factor in exercise adherence (20). Hydari et al. (21) also reported that gamification elements applied to wearable devices positively contribute to increased exercise participation, with greater effects observed among users who had previously exhibited lower levels of physical activity. Furthermore, qualitative evidence suggests that wearable device users may experience internal conflict, highlighting that the psychological effects of wearable devices are highly individualized and cannot be fully captured through quantitative approaches alone (22).

Long-term longitudinal studies targeting new sport participants have repeatedly reported difficulties in sustaining exercise adherence. Gjestvang et al. (23) conducted a one-year follow-up study of new exercisers and found that the proportion of participants who continued exercising at least twice per week was approximately 51.8% at the 3-month mark, declining further to 37.6% at 6 months and 37.4% at 12 months. Notably, only approximately 17% of participants maintained regular exercise throughout the entire study period, indicating that a substantial proportion of new sport participants either dropped out or exhibited irregular participation patterns during their engagement. These findings suggest that dropout and irregular participation are highly prevalent phenomena among new sports participants.

These prior studies suggest that wearable devices can serve as effective tools for reducing the likelihood of dropout and enhancing exercise adherence among new sport participants. That is, wearable devices not only produce behavioral changes but also play a multifaceted role in promoting sustained exercise participation by interacting with psychological factors such as social interaction, goal setting, and the enhancement of self-efficacy. However, empirical research verifying these psychological and social mechanisms has been conducted primarily in international contexts and remains limited within South Korea. In particular, studies examining the effects of wearable devices with a focus on the early psychological changes and exercise continuation intentions of new sport participants are largely absent from the existing literature. Furthermore, the potential public mental health implications of wearable device use among new sport participants—particularly with respect to motivation, psychological engagement, and dropout—have yet to be systematically examined.

The present study draws on three complementary theoretical frameworks to explain how wearable device use may shape the psychological experiences of new sport participants. First, self-determination theory [SDT; (24)] conceptualizes motivation along a continuum ranging from intrinsic motivation through extrinsic motivation to amotivation, and posits that the satisfaction of three basic psychological needs—autonomy, competence, and relatedness—fosters more autonomous forms of motivation. The self-monitoring, goal-setting, and feedback functions of wearable devices may serve as external supports for competence and autonomy, whereas the data sharing function may address the need for relatedness. Second, flow theory (25) proposes that flow arises when a balance is maintained between perceived challenge and skill, and that clear goals and immediate feedback are key preconditions of flow—conditions that wearable devices are structurally well suited to provide (26). Third, the theory of planned behavior (27) suggests that attitudes toward a behavior shape behavioral intentions, implying that the positive attitudes and psychological engagement fostered by wearable device use may translate into lower intentions to discontinue sport participation. Within this integrated framework, the following empirical evidence provides the basis for the research hypotheses.

From an SDT perspective, wearable devices may function as external facilitators of basic psychological need satisfaction, thereby promoting more self-determined forms of motivation. Wearable device use can enhance exercise motivation and accountability, as well as strengthen goal-pursuit motivation (28, 29). Furthermore, perceived competence is significantly associated with the intention to use wearable devices (30), and exercise motivation is closely related to adherence to physical activity.

Consistent with cognitive evaluation theory (31), feedback that is perceived as informational rather than controlling can foster positive evaluations of an activity. Wearable device use can influence the formation of positive perceptions and attitudes toward physical activity (32). Furthermore, device use can positively affect exercise motivation and physical activity adherence (29) and has been associated with an increased likelihood of engaging in recommended levels of physical activity and meeting established guidelines (33, 34). Wearable devices exert positive influences not only on behavioral outcomes but also on attitudinal dimensions.

Given that clear goals and immediate feedback constitute key antecedents of flow (25), the real-time feedback provided by wearable devices may be particularly conducive to flow experiences. Wearable devices can enhance the flow experience during exercise, and flow can exert a significant influence on exercise participation intention (35). Furthermore, the possibility of quantitatively analyzing flow states through physiological signal-based measurements has been proposed (36). Additionally, gamification, challenge, and mastery factors within wearable device use environments contribute to the enhancement of flow (37, 38).

In line with the theory of planned behavior, positive attitudes and sustained engagement fostered by device use may reduce the intention to discontinue participation. Exercise interventions utilizing wearable devices can yield high levels of participation adherence and low dropout rates, and wearable device use positively contributes to suppressing the decline in physical activity levels over time (39, 40). Furthermore, wearable devices enhance autonomous motivation, thereby strengthening exercise adherence and ultimately reducing dropout (41).

Therefore, the purpose of this study is to analyze the effects of wearable device use and utilization patterns on psychological factors—including self-regulatory motivation, flow, participation attitude, and dropout—among new sport participants, and to provide foundational data for promoting sustained participation among individuals newly engaged in sport activities. To address this gap, the present study categorizes participants into three groups based on their wearable device usage patterns—the sharing-and-using group, the using-only group, and the non-using group—thereby allowing for a more nuanced examination of how different modes of device utilization influence psychological outcomes.

### 1.1 Research hypotheses

Hypothesis 1: There will be significant group differences in self-regulatory motivation according to wearable device usage patterns (i.e., the sharing-and-using group, the using-only group, and the non-using group).

Hypothesis 2: There will be significant group differences in participation attitude according to wearable device usage patterns (i.e., the sharing-and-using group, the using-only group, and the non-using group).

Hypothesis 3: There will be significant group differences in flow according to wearable device usage patterns (i.e., the sharing-and-using group, the using-only group, and the non-using group).

Hypothesis 4: There will be significant group differences in dropout according to wearable device usage patterns (i.e., the sharing-and-using group, the using-only group, and the non-using group).

## 2 Methodology

### 2.1 Study participants

To achieve the purpose of this study, survey data were collected from new sport participants through an online platform; these participants were subsequently selected as the sample group. In this study, new sport participants were operationally defined as individuals who had been participating in sport activities for < 12 months. This criterion was based on Gjestvang et al. (23), who reported that only approximately 17% of new fitness members achieved regular exercise behavior during their first year of membership, indicating that the first 12 months represent a critical period for exercise adherence. Accordingly, participation duration was categorized into five intervals: < 1 month, 1–3 months, 4–6 months, 7–9 months, and 10–12 months. Sampling was conducted using a convenience sampling method, which is one of the non-probability sampling methods. Convenience sampling was deemed appropriate for the present study because new sport participants—individuals within their first 12 months of participation—constitute a transient and hard-to-reach population for which no sampling frame exists, making probability sampling practically infeasible. Online recruitment via social networking services was employed to reach this population efficiently across diverse sport types and regions. Data collection was performed online following institutional review board approval (No. KHGIRB-25-712). Participants were recruited through social networking services (SNS), and survey links were distributed using online Google Forms. No compensation was provided for participation, and the questionnaire took approximately 3 min to complete.

Participants were male and female adults aged 20 years or older residing in South Korea, with a focus on new sport participants. In this study, wearable devices were defined as devices that automatically record and store physical activity or health-related data, including smartwatches, smart bands, and fitness trackers. Participants were classified into three groups based on two survey items assessing wearable device usage and data sharing behavior: the sharing-and-using group (those who used a wearable device and shared their exercise records with others), the using-only group (those who used a wearable device but did not share their records), and the non-using group (those who did not use a wearable device). Eligibility was verified through a screening item assessing the duration of sport participation, and only respondents reporting < 12 months of participation were included in the analysis. A total of 256 questionnaires were collected. Of these, 30 were excluded based on two criteria: identical responses across all items (straight-lining) and failure to complete the questionnaire. This resulted in a final sample of 226 valid questionnaires, and as incomplete responses were excluded at this stage, the final dataset contained no missing data.

Demographic characteristics were assessed using eight items, including sex, age, sport type participated in, duration of sport participation, frequency of sport activity participation, wearable device usage, method of wearable device utilization (including whether data were shared), and type of wearable device used. The specific demographic characteristics of the participants are presented in Table 1.

**Table 1:** Descriptive statistics.

Characteristic	Comparison	Group 1	Group 2	Group 3
		*n* (%)	*n* (%)	*n* (%)
Sex	Male	44 (57.9%)	34 (47.9%)	47 (59.5%)
	Female	32 (42.1%)	37 (52.1%)	32 (40.5%)
Age (years)	20 s	26 (34.2%)	24 (33.8%)	37 (46.8%)
	30 s	16 (21.1%)	13 (18.3%)	6 (7.6%)
	40 s	18 (23.7%)	21 (29.6%)	19 (24.1%)
	Over 50 s	16 (21.1%)	13 (18.3%)	17 (21.5%)
Duration of participation	< 1 month	11 (14.5%)	12 (16.9%)	16 (20.3%)
	1–3 months	17 (22.4%)	11 (15.5%)	3 (3.8%)
	4–6 months	17 (22.4%)	14 (19.7%)	17 (21.5%)
	7–9 months	8 (10.5%)	8 (11.3%)	7 (8.9%)
	10–12 months	23 (30.3%)	26 (36.6%)	36 (45.6%)
Frequency of participation	Less than once a month	3 (3.9%)	3 (4.2%)	14 (17.7%)
	1–2 times a month	13 (17.1%)	9 (12.7%)	15 (19.0%)
	Once a week	17 (22.4%)	21 (29.6%)	11 (13.9%)
	Twice or more per week	36 (47.4%)	29 (40.8%)	28 (35.4%)
	Almost every day	7 (9.2%)	9 (12.7%)	11 (13.9%)
	Total	76 (33.6%)	71 (31.4%)	79 (35.0%)

Group 1 = sharing-and-using group, Group 2 = using-only group, Group 3 = non-using group.

### 2.2 Instruments

Participants completed the questionnaire using a self-administration method. All measurement instruments were adapted from previously validated scales whose reliability and validity have been established in prior studies (27, 42–45), and the validity and reliability of the adapted instruments were further verified in the present study through EFA and Cronbach's α (see Section 3.1). The questionnaire consisted of 34 items, comprising eight items on demographic characteristics, nine items on self-regulatory motivation, six items on participation attitude, seven items on flow, and four items on dropout. Each item was measured on a 5-point Likert scale: “strongly disagree” (1), “disagree” (2), “neutral” (3), “agree” (4), and “strongly agree” (5).

#### 2.2.1 Self-regulatory motivation

Self-regulatory motivation, one of the dependent variables examined to analyze differences according to the independent variable, was measured using the Sport Motivation Scale originally developed by Pelletier et al. (42), translated and validated by Mun et al. (43), and subsequently modified and supplemented to align with the purpose of this study. Self-regulatory motivation consisted of three sub-factors—intrinsic motivation, extrinsic motivation, and amotivation—with three items per sub-factor, yielding nine items.

#### 2.2.2 Participation attitude

Participation attitude was measured using a scale adapted and modified from the Leisure Attitude Scale developed by Ragheb and Beard (44), adjusted to fit the purpose of this study. Participation attitude consisted of two sub-factors—proactiveness and perseverance—with three items each, yielding six items.

#### 2.2.3 Flow

Flow was measured using items translated by Jung (46) based on the questionnaire developed by Scanlan et al. (45), which were subsequently modified and restructured to suit the purpose of this study. Flow consisted of two sub-factors—cognitive flow and behavioral flow—comprising four items for cognitive flow and three items for behavioral flow, yielding seven items.

#### 2.2.4 Dropout

Dropout was measured using the intention items from the Theory of Planned Behavior scale developed by Ajzen and Driver (27), with reference to items used in the study by Yoo and Kim (47), modified and supplemented to align with the purpose of this study. Dropout consisted of a single factor comprising four items. It should be noted that dropout in the present study was operationalized as dropout intention rather than actual dropout behavior, reflecting the participants' self-reported likelihood of discontinuing sport participation.

### 2.3 Data analysis

All data in this study were processed using SPSS 29.0. First, frequency analysis was conducted to identify the demographic characteristics of new sport participants according to wearable device usage patterns (sharing-and-using group, using-only group, and non-using group). Second, exploratory factor analysis (EFA) was conducted to verify the validity of the collected questionnaires. The factor analysis employed the Kaiser–Meyer–Olkin (KMO) measure, Bartlett's test of sphericity, the principal component extraction method, and Varimax orthogonal rotation. Cronbach's α reliability coefficients were subsequently used to assess the reliability of the measurement instruments. Finally, correlation analysis was conducted to examine the relationships among the factors, and Multivariate Analysis of Variance (MANOVA) with *post-hoc* analysis was performed to compare differences in self-regulatory motivation, participation attitude, flow, and dropout according to wearable device usage patterns among new sport participants.

## 3 Results

### 3.1 Validity and reliability

The results of the validity analysis for self-regulatory motivation indicated a KMO value of 0.794 and a Bartlett's test *p*-value of < 0.001, confirming that the data were appropriate for EFA. Three sub-factors were extracted: intrinsic motivation, extrinsic motivation, and amotivation. Subsequent reliability analysis yielded Cronbach's alpha coefficients of 0.927 for intrinsic motivation, 0.621 for extrinsic motivation, and 0.865 for amotivation, thereby establishing acceptable reliability for the measurement instrument (Table 2). Although the reliability of extrinsic motivation was relatively low, Cronbach's alpha values exceeding 0.6 are considered acceptable (48). Furthermore, alpha values can vary depending on the number of items, the dimensionality of the construct, and sample characteristics (49), and scales with alpha below 0.70 should be evaluated in light of the research context rather than being discarded (48). Given that extrinsic motivation consisted of only three items, the obtained value was deemed acceptable for the present study.

**Table 2:** Exploratory factor analysis and Cronbach's alpha reliability results for self-regulatory motivation.

Items	1	2	3
Intrinsic motivation 3	**0.909**	−0.211	0.029
Intrinsic motivation 1	**0.897**	−0.254	0.087
Intrinsic Motivation 2	**0.892**	−0.222	0.073
Amotivation 2	−0.256	**0.889**	0.026
Amotivation 1	−0.177	**0.872**	0.061
Amotivation 3	−0.185	**0.833**	−0.029
Extrinsic motivation 1	0.046	0.020	**0.815**
Extrinsic motivation 3	−0.081	0.059	**0.791**
Extrinsic motivation 2	0.398	−0.056	**0.619**
Eigenvalue	3.790	1.825	1.211
Variance (%)	42.113	20.280	13.453
Cronbach's alpha	0.927	0.865	0.621

KMO = 0.794, χ^2^ = 1,070.065, df = 36, *p* < 0.001. Bold values indicate the primary factor loading of each item on its extracted factor.

The results of the validity analysis for participation attitude indicated a KMO value of 0.815 and a Bartlett's test *p*-value of < 0.001, confirming that the data were appropriate for EFA. Two sub-factors were extracted: proactiveness and perseverance. Subsequent reliability analysis yielded Cronbach's alpha coefficients of 0.918 for proactiveness and 0.892 for perseverance, with all factors exceeding the threshold value of 0.700, thereby establishing acceptable reliability for the measurement instrument (Table 3).

**Table 3:** Exploratory factor analysis and Cronbach's alpha reliability results for participation attitude.

Items	1	2
Proactiveness 2	**0.918**	0.213
Proactiveness 1	**0.909**	0.266
Proactiveness 3	**0.845**	0.294
Perseverance 2	0.199	**0.892**
Perseverance 3	0.256	**0.865**
Perseverance 1	0.306	**0.855**
Eigenvalue	3.877	1.182
Variance (%)	64.619	19.702
Cronbach's alpha	0.918	0.892

KMO = 0.815, χ^2^ = 996.853, df = 15, *p* < 0.001. Bold values indicate the primary factor loading of each item on its extracted factor.

The results of the validity analysis for flow indicated a KMO value of 0.868 and a Bartlett's test *p*-value of < 0.001, confirming that the data were appropriate for EFA. Two sub-factors were extracted: cognitive flow and behavioral flow. Subsequent reliability analysis yielded Cronbach's alpha coefficients of 0.909 for cognitive flow and 0.868 for behavioral flow, with all factors exceeding the threshold value of 0.700, thereby establishing acceptable reliability for the measurement instrument (Table 4).

**Table 4:** Exploratory factor analysis and Cronbach's alpha reliability results for flow.

Items	1	2
Cognitive flow 3	**0.863**	0.292
Cognitive flow 4	**0.854**	0.308
Cognitive flow 2	**0.838**	0.266
Cognitive flow 1	**0.832**	0.188
Behavioral flow 2	0.213	**0.877**
Behavioral flow 1	0.276	**0.849**
Behavioral flow 3	0.297	**0.828**
Eigenvalue	4.362	1.179
Variance (%)	62.313	16.837
Cronbach's alpha	0.909	0.868

KMO = 0.868, χ^2^ = 1044.400, df = 21, *p* < 0.001. Bold values indicate the primary factor loading of each item on its extracted factor.

The results of the validity analysis for dropout indicated a KMO value of 0.777 and a Bartlett's test *p*-value of < 0.001, confirming that the data were appropriate for EFA. Dropout was confirmed as a unidimensional factor, and subsequent reliability analysis yielded a Cronbach's alpha coefficient of 0.852, exceeding the threshold value of 0.700, thereby establishing acceptable reliability for the measurement instrument (Table 5).

**Table 5:** Exploratory factor analysis and Cronbach's alpha reliability results for dropout.

Items	1
Dropout 4	0.902
Dropout 2	0.872
Dropout 3	0.827
Dropout 1	0.719
Eigenvalue	2.776
Variance (%)	69.397
Cronbach's alpha	0.852

KMO = 0.777 χ^2^ = 425.321, df = 6, *p* < 0.001.

### 3.2 Pearson correlation analysis

To examine the relationships among the variables, a Pearson correlation analysis was conducted. The results indicated that most variables were significant, and all correlation coefficients were below 0.80, suggesting that multicollinearity was not a concern in this study (Table 6).

**Table 6:** Pearson correlation analysis among dependent variables for multicollinearity.

Variable	1	2	3	4	5	6	7	8
1	1							
2	0.192^**^	1						
3	−0.459^**^	0.003	1					
4	0.563^**^	0.276^**^	−0.496^**^	1				
5	0.593^**^	0.116	−0.430^**^	0.537^**^	1			
6	0.557^**^	0.232^**^	−0.460^**^	0.636^**^	0.590^**^	1		
7	0.520^**^	0.272^**^	−0.359^**^	0.571^**^	0.529^**^	0.564^**^	1	
8	−0.455^**^	−0.040	0.528^**^	−0.425^**^	−0.472^**^	−0.434^**^	−0.288^**^	1

^**^p < 0.01; 1 = Intrinsic Motivation, 2 = Extrinsic Motivation, 3 = Amotivation, 4 = Proactiveness, 5 = Perseverance, 6 = Cognitive Flow, 7 = Behavioral Flow, 8 = Dropout.

### 3.3 MANOVA and *post-hoc* analysis

To examine whether differences existed in self-regulatory motivation, participation attitude, flow, and dropout according to wearable device usage patterns among new sport participants, a MANOVA was conducted. First, Box's test of equality of covariance matrices was performed to verify the homogeneity of covariance matrices: Box's *M* = 156.114, *F* = 2.058, *p* < 0.001, indicating a violation of this assumption. Because Box's test is known to be highly sensitive to sample size and non-normality (50), and because Pillai's Trace is more robust than Wilks‘ Lambda under violations of this assumption—particularly when group sizes are approximately equal, as in the present study (*n* = 76, 71, and 79)—both multivariate criteria were examined. The multivariate effect was significant by both criteria (Wilks' Λ = 0.845, *F*(16, 432) = 2.364, *p* = 0.002, partial η^2^ = 0.081; Pillai's Trace = 0.158, *F*(16, 434) = 2.321, *p* = 0.003, partial η^2^ = 0.079), indicating that the significance of the overall multivariate effect was not contingent on the choice of test statistic. Levene's tests indicated homogeneity of error variances for all dependent variables except extrinsic motivation and behavioral flow; accordingly, the conservative Scheffé procedure was employed for *post-hoc* comparisons. Nevertheless, the results are interpreted with these assumption violations in mind. Subsequent univariate analyses revealed significant group differences for most dependent variables. The variables that demonstrated significant differences were as follows: (a) intrinsic motivation (*F* = 8.634, *p* < 0.001), (b) extrinsic motivation (*F* = 3.572, *p* = 0.030), (c) amotivation (*F* = 7.428, *p* < 0.001), (d) perseverance (*F* = 5.275, *p* = 0.006), (e) cognitive flow (*F* = 6.966, *p* = 0.001), (f) behavioral flow (*F* = 6.367, *p* = 0.002), and (g) dropout (*F* = 6.547, *p* = 0.002). Proactiveness did not reach statistical significance (*p* = 0.067; Table 7).

**Table 7:** Multivariate analysis of variance results by wearable device usage patterns.

Variable	Sub-factor	df	*F*	*p*	*η^2^*	Scheffé
Self-regulatory motivation	Intrinsic	2	8.634	< 0.001	0.072	a > c, b > c
	Extrinsic	2	3.572	0.030	0.031	a > c, b > c
	Amotivation	2	7.428	< 0.001	0.062	a < c, b < c
Participation attitude	Proactiveness	2	2.736	0.067	0.024	-
	Perseverance	2	5.275	0.006	0.045	a > c, b > c
Flow	Cognitive	2	6.966	0.001	0.059	a > c, b > c
	Behavioral	2	6.367	0.002	0.054	a > c, b > c
Dropout		2	6.547	0.002	0.055	b < c

a = sharing-and-using group, b = using-only group, c = non-using group.

The effect sizes for the significant variables were generally small to moderate. Specifically, intrinsic motivation yielded the largest effect (partial η^2^ = 0.072), followed by amotivation (partial η^2^ = 0.062), cognitive flow (partial η^2^ = 0.059), dropout (partial η^2^ = 0.055), behavioral flow (partial η^2^ = 0.054), perseverance (partial η^2^ = 0.045), and extrinsic motivation (partial η^2^ = 0.031). According to Cohen's (51) conventions, partial η^2^ values of 0.01, 0.06, and 0.14 represent small, medium, and large effects, respectively. These results suggest that while the group differences were statistically significant, the magnitude of the effects was relatively modest, which should be considered when interpreting the practical significance of the findings.

Since this study analyzed differences among three wearable device usage pattern groups, a *post-hoc* analysis was conducted to identify the specific nature of the significant group differences. For intrinsic motivation and extrinsic motivation, both sub-factors of self-regulatory motivation, the sharing-and-using group scored significantly higher than the non-using group, and the using-only group also scored significantly higher than the non-using group. In terms of mean scores, the sharing-and-using group (*M* = 4.36) and the using-only group (*M* = 4.35) demonstrated notably higher intrinsic motivation than the non-using group (*M* = 3.93; Table 8). For amotivation, the sharing-and-using group scored significantly lower than the non-using group, and the using-only group also scored significantly lower than the non-using group. The non-using group reported the highest amotivation (*M* = 1.97), compared to the sharing-and-using group (*M* = 1.57) and the using-only group (*M* = 1.57; Table 8). Concerning participation attitude, perseverance showed that the sharing-and-using group scored significantly higher than the non-using group, and the using-only group also scored significantly higher than the non-using group. No significant group differences were found for proactiveness. Regarding flow, both sub-factors—cognitive flow and behavioral flow—indicated that the sharing-and-using group scored significantly higher than the non-using group, and the using-only group also scored significantly higher than the non-using group. Mean scores for cognitive flow were 4.22, 4.20, and 3.82 for the sharing-and-using, using-only, and non-using groups, respectively (Table 8). Finally, while dropout showed a significant overall group difference (*F* = 6.547, *p* = 0.002), *post-hoc* analysis revealed that only the using-only group demonstrated significantly lower dropout levels than the non-using group (*M* = 1.85 vs. *M* = 2.35), whereas no significant difference was found between the sharing-and-using group (*M* = 2.10) and the non-using group (Table 8; Table 9).

**Table 8:** Mean score differences by wearable device usage patterns.

Group	Self-regulatory motivation			Participation attitude		Flow		Dropout
	1	2	3	4	5	6	7	
G1	4.3596	3.1711	1.5658	4.1053	4.1491	4.2171	3.9693	2.1020
G2	4.3474	3.2019	1.5681	4.1033	4.1925	4.1972	3.9296	1.8521
G3	3.9283	2.8987	1.9747	3.7975	3.7890	3.8165	3.5105	2.3481

G1 = sharing-and-using group, G2 = using-only group, G3 = non-using group; 1 = intrinsic motivation, 2 = extrinsic motivation, 3 = amotivation, 4 = proactiveness, 5 = perseverance, 6 = cognitive flow, 7 = behavioral flow.

**Table 9:** *Post-hoc* analysis results by wearable device usage patterns.

Group	Comparison	Self-regulatory motivation			Participation attitude		Flow		Dropout
		1	2	3	4	5	6	7	
G1	G2	0.995	0.971	1.000	1.000	0.953	0.987	0.964	0.198
	G3	0.002^**^	0.093	0.004^**^	0.128	0.031^*^	0.005^**^	0.006^**^	0.191
G2	G1	0.995	0.971	1.000	1.000	0.953	0.987	0.964	0.198
	G3	0.003^**^	0.059	0.005^**^	0.141	0.015^*^	0.009^**^	0.016^*^	0.002^**^
G3	G1	0.002^**^	0.093	0.004^**^	0.128	0.031^*^	0.005^**^	0.006^**^	0.191
	G2	0.003^**^	0.059	0.005^**^	0.141	0.015^*^	0.009^**^	0.016^*^	0.002^**^

^**^p < 0.01, ^*^p < 0.05; G1 = sharing-and-using group, G2 = using-only group, G3 = non-using group; 1 = intrinsic motivation, 2 = extrinsic motivation, 3 = amotivation, 4 = proactiveness, 5 = perseverance, 6 = cognitive flow, 7 = behavioral flow.

## 4 Discussion

This study conducted a comparative analysis of self-regulatory motivation, participation attitude, flow, and dropout based on wearable device use and utilization patterns among new sport participants. The following discussion interprets the findings in relation to prior research and existing theoretical frameworks, while acknowledging the cross-sectional nature of the study, which precludes causal inferences.

First, concerning self-regulatory motivation, the wearable device usage groups (sharing-and-using and using-only) demonstrated higher levels of intrinsic motivation and extrinsic motivation, and lower levels of amotivation compared to the non-using group. Although causal relationships cannot be established given the cross-sectional design, these findings are consistent with the notion that wearable devices function not merely as recording tools but as psychological mechanisms that facilitate self-regulatory motivation. The effect sizes for intrinsic motivation (partial η^2^ = 0.072) and amotivation (partial η^2^ = 0.062) were in the medium range, suggesting meaningful practical differences among the groups. In particular, Steel (52) reported that wearable technology use is positively associated with both autonomous motivation and physical activity levels, and that this relationship is maintained over time. Furthermore, Kerner and Goodyear (53) reported that wearable devices positively influence motivational regulation by supporting the satisfaction of basic psychological needs, including autonomy, competence, and relatedness. These findings align with self-determination theory (24), which posits that satisfaction of basic psychological needs is essential for fostering autonomous motivation, suggesting that wearable devices may serve as external facilitators of need satisfaction in sport participation contexts.

Second, the wearable device usage groups (sharing-and-using and using-only) demonstrated higher levels of participation attitude compared to the non-using group. While causal inferences cannot be drawn, these findings suggest that wearable devices contribute to the formation and maintenance of positive perceptions of exercise. Wearable device users continuously monitor their exercise performance and receive real-time feedback on metrics such as activity levels and goal attainment, thereby accumulating experiences of achievement. This process serves as a factor that fosters positive perceptions of exercise and reinforces participation attitude. With respect to effect sizes, perseverance yielded a small-to-medium effect (partial η^2^ = 0.045), while proactiveness did not reach statistical significance (*p* = 0.067), suggesting that the influence of wearable devices on participation attitude may be more pronounced for perseverance than for proactiveness. These findings are consistent with prior research. Brickwood et al. (54) reported that wearable activity trackers promote behavioral change through self-monitoring and feedback, suggesting that users can positively shift their attitudes toward exercise participation by recognizing and regulating their own behavior. Furthermore, a systematic review of systematic reviews (19) demonstrated that wearable activity trackers directly enhance users' attitudes toward physical activity and their levels of participation, emphasizing that wearable devices extend beyond simple behavioral change tools to influence cognitive and affective attitude formation. These findings can be further understood through the lens of cognitive evaluation theory (31), which suggests that external feedback that is perceived as informational rather than controlling can enhance intrinsic motivation and positive attitudes toward an activity.

Third, the wearable device usage groups (sharing-and-using and using-only) demonstrated higher levels of flow compared to the non-using group. While causal relationships cannot be established, these findings suggest that the real-time feedback, goal achievement notifications, and gamification elements provided by wearable devices enhance concentration during exercise. In terms of effect sizes, cognitive flow (partial η^2^ = 0.059) and behavioral flow (partial η^2^ = 0.054) both approached medium effect sizes, indicating meaningful practical differences among the groups. These results are consistent with prior research on gamification elements. Johnson et al. (55) reported that gamification elements such as feedback, goal setting, and rewards are key factors that increase user engagement and flow, which is closely aligned with the functional characteristics offered by wearable devices. That is, wearable devices extend beyond simple measurement tools to transform the exercise environment into a gamified context, thereby enhancing users' flow experience. Furthermore, given that flow experience is formed when a balance is maintained between an individual's skill level and the level of challenge presented by a task (26), data-driven feedback provided by wearable devices plays an important role in facilitating flow formation. Considering that external stimuli play a significant role in the initial formation of flow, these findings suggest that wearable devices can function as effective tools for promoting flow in the early stages of exercise participation. These findings extend Csikszentmihalyi (25) flow theory by demonstrating that technologically mediated feedback may serve as an external trigger for flow states, particularly among new sport participants who are still developing their skill levels.

Fourth, differential results were observed among the groups concerning dropout. The analysis revealed that the using-only group demonstrated significantly lower levels of dropout compared to the non-using group; however, no significant difference was found between the sharing-and-using group and the non-using group. It should be noted that dropout in the present study was operationalized as dropout intention rather than actual dropout behavior, and the following discussion should be interpreted accordingly. Furthermore, while causal relationships cannot be established, these findings suggest that while wearable device use itself contributes to enhancing exercise adherence, the additional function of data sharing does not necessarily translate into further reductions in dropout. The mere act of monitoring and recording one's own physical activity data through a wearable device appears sufficient to support exercise continuation. In contrast, the social dimension introduced by data sharing may not consistently provide additional benefits in reducing dropout among new sport participants. The effect size for dropout was small-to-medium (partial η^2^ = 0.055), suggesting that while the group difference was statistically significant, the practical magnitude of the effect warrants cautious interpretation. This pattern of results implies that the relationship between wearable device utilization and dropout is more nuanced than a simple linear association, and that the specific mode of device use—rather than use alone—plays a meaningful role in shaping behavioral outcomes related to exercise adherence.

These results can be explained by the dual characteristics of the wearable device's sharing function. When individuals use a wearable device, they focus on their own data to engage in self-regulation and goal management, which helps strengthen exercise adherence. In contrast, when data are shared with others, exercise performance may shift from a personal activity to one involving social comparison and evaluation. According to prior research, data sharing via wearable devices induces social comparison among users, and comparing oneself with others who demonstrate higher performance levels can lead to frustration and negative affect, which may subsequently result in reduced device use (28). Similarly, a meta-review of social comparison features in physical activity apps concluded that the motivational effects of comparison are inconsistent and can be demotivating for some users (56). Furthermore, data sharing in online environments can reinforce the desire to present oneself positively to others while simultaneously increasing anxiety and psychological burden through social comparison (57). In particular, new sport participants who are in the early stages of forming stable exercise habits may be more vulnerable to social comparison and evaluation. Consequently, when psychological pressure accumulates during the exercise process, it may impede continuation and ultimately contribute to dropout. These findings contrast with Chang et al. (20), who reported that data sharing via wearable devices promotes social support and increases exercise adherence, suggesting that the effect of data sharing may vary depending on individual characteristics and exercise experience level. Data sharing thus possesses a dual nature: it can facilitate social support and motivational enhancement, while simultaneously weakening exercise adherence through the burden of self-presentation and social evaluation. Given that preferences for social interaction in wearable tracker use vary considerably across individuals (58), the value of the sharing function appears contingent on individual dispositions. From the perspective of cognitive evaluation theory (31), shared data may satisfy the need for relatedness when perceived as informational feedback, but may undermine autonomous motivation when experienced as controlling through social comparison.

The present findings offer several practical implications. Fitness facilities may incorporate wearable devices into onboarding programs for novice exercisers, whose dropout risk is highest during the first year of participation. Device and application developers should consider designing data sharing as an optional (opt-in) feature and centering feedback for novices on self-referenced progress rather than comparison with others, while public health practitioners may utilize wearable devices as low-cost, scalable tools in community-based programs for individuals in the early stages of exercise adoption.

## 5 Conclusions and future research

This study examined differences in self-regulatory motivation, participation attitude, flow, and dropout according to wearable device usage patterns (sharing-and-using, using-only, and non-using groups) among new sport participants. It should be noted that dropout was measured as intention rather than actual behavior, which should be considered when generalizing the findings. The findings confirmed that wearable devices serve as effective tools for positively influencing psychological factors—including self-regulatory motivation, participation attitude, and flow—among new sport participants. However, concerning behavioral outcomes such as dropout, the effects varied depending on the mode of device utilization, and the data sharing function in particular demonstrated a dual nature, yielding both positive and negative effects simultaneously. These findings contribute to the literature by extending self-determination theory and flow theory to the context of wearable device use among new sport participants, demonstrating that technologically mediated feedback can serve as an external facilitator of psychological engagement in sport participation. These results suggest that maximizing the effectiveness of wearable devices requires not merely promoting their distribution or general use, but rather strategic utilization that considers individual user characteristics, exercise experience, and personal goals. Based on the findings of this study, the following recommendations are proposed.

First, the cross-sectional nature of this study limits the ability to establish causal relationships between wearable device use and psychological outcomes. Future research should employ longitudinal study designs to more rigorously examine the long-term effects of wearable device use on exercise adherence. A longitudinal approach would allow researchers to track changes in self-regulatory motivation, participation attitude, flow, and dropout at multiple time points, thereby providing a more comprehensive understanding of how wearable device use influences the psychological and behavioral trajectories of new sport participants. Furthermore, future studies should consider a range of individual difference variables—including age, sex, sport type, and personality traits—to better account for the heterogeneity among participants and to identify subgroup-specific effects of wearable device use. Furthermore, the use of convenience sampling through online recruitment introduces potential selection bias, as digitally engaged individuals may have been overrepresented, and eligibility relied on self-reported participation duration. These characteristics limit the generalizability of the findings.

Additionally, the present study did not control for potential confounding variables such as prior exercise experience, socioeconomic status, and access to technology, which may have influenced the observed group differences. Future research should incorporate appropriate controls for such variables to enhance the internal validity of the findings. In addition, although EFA was employed in the present study because the measurement instruments were modified and applied to a new population—in which case the factor structure cannot be assumed to replicate the original scales (59)—confirmatory factor analysis (CFA) was not conducted, as performing both EFA and CFA on the same sample raises concerns of capitalization on chance, and the sample size did not permit a split-sample approach. Future research should cross-validate the factor structure identified in this study through CFA with an independent sample.

Second, this study was limited in its ability to fully account for the psychological mechanisms underlying the observed group differences. Future research should incorporate moderating variables such as individual personality traits, social comparison orientation, and exercise experience level, as these may play a significant role in determining the extent to which wearable device use and data sharing influence motivational and behavioral outcomes. In particular, given the dual nature of the data sharing function identified in this study, future research should more closely examine the conditions under which data sharing produces positive vs. negative effects. Variables such as self-esteem, competitive disposition, and digital literacy may moderate the relationship between data sharing and exercise adherence, and their inclusion would contribute to a more nuanced theoretical understanding of wearable device use in sport and physical activity contexts. Additionally, future studies may benefit from incorporating qualitative methods to capture the subjective experiences of new sport participants, which would complement the quantitative findings of this study and provide deeper insights into the psychological processes involved.

## Data Availability

The original contributions presented in the study are included in the article/supplementary material, further inquiries can be directed to the corresponding author/s.
